# Adaptation of spatio‐temporal convergent properties in central vestibular neurons in monkeys

**DOI:** 10.14814/phy2.13750

**Published:** 2018-09-03

**Authors:** Julia N. Eron, Dmitri Ogorodnikov, Anja K. E. Horn, Sergei B. Yakushin

**Affiliations:** ^1^ Department of Neurology Icahn School of Medicine at Mount Sinai New York New York; ^2^ FNND LLC Elmwood Park New Jersey; ^3^ Institute of Anatomy and Cell Biology Ludwig‐Maximilians‐Universität Munich Germany

**Keywords:** Canal–otolith and otolith‐only neurons, four‐component model, orientation adaptation, regular and irregular vestibular inputs, spatio‐temporal convergence

## Abstract

The spatio‐temporal convergent (STC) response occurs in central vestibular cells when dynamic and static inputs are activated. The functional significance of STC behavior is not fully understood. Whether STC is a property of some specific central vestibular neurons, or whether it is a response that can be induced in any neuron at some frequencies is unknown. It is also unknown how the change in orientation of otolith polarization vector (orientation adaptation) affects STC behavior. A new complex model, that includes inputs with regular and irregular discharges from both canal and otolith afferents, was applied to experimental data to determine how many convergent inputs are sufficient to explain the STC behavior as a function of frequency and orientation adaptation. The canal–otolith and otolith‐only neurons were recorded in the vestibular nuclei of three monkeys. About 42% (11/26 canal–otolith and 3/7 otolith‐only) neurons showed typical STC responses at least at one frequency before orientation adaptation. After orientation adaptation in side‐down head position for 2 h, some canal–otolith and otolith‐only neurons altered their STC responses. Thus, STC is a property of weights of the regular and irregular vestibular afferent inputs to central vestibular neurons which appear and/or disappear based on stimulus frequency and orientation adaptation. This indicates that STC properties are more common for central vestibular neurons than previously assumed. While gravity‐dependent adaptation is also critically dependent on stimulus frequency and orientation adaptation, we propose that STC behavior is also linked to the neural network responsible for localized contextual learning during gravity‐dependent adaptation.

## Introduction

Natural head movements activate all three pairs of the semicircular canals and the otolith organs in the vestibular labyrinths, and then the vestibular afferent signals transform and process in vestibular nuclei (VN) during both rotational and translational motions and tilts. Some neurons in VN have spatio‐temporal convergence (STC) behavior which arises from the convergence of vestibular inputs with different spatial and temporal tuning properties (Baker et al. 1984a,b; Kasper et al. 1988; Angelaki et al. 1992b; Bush et al. 1993).

The neurons with STC response have firing rates (FR) that are modulated with sinusoidal head rotations about a spatial horizontal axis in every head orientation in yaw. Their temporal phases monotonically change from being close to head position to being in‐phase with head velocity as yaw head orientation is changed relative to the direction of tilt (Curthoys and Markham 1971; Daunton and Melvill‐Jones 1982; Baker et al. 1984a,b; Schor et al. 1984; Kasper et al. 1988; Yakushin et al. 1999), thereby those responses indicate an interaction of canal and otolith sensory inputs. In contrast to STC behavior, the temporal phases of non‐STC behavior are fixed relative to either head position or velocity in each head orientation, and there are no response in one orientation and maximal response in an orthogonal head orientation in yaw. STC characteristics have also been induced by linear acceleration in the horizontal plane (Schor et al. 1984; Angelaki 1992a, 1993; Bush et al. 1993; Kleine et al. 1999; Angelaki and Dickman 2000; Dickman and Angelaki 2002) as well as in 3‐D (Chen‐Huang and Peterson 2006, 2010), indicating that static and dynamic otolith inputs are sufficient source of STC.

The frequency dependence of STC properties in pure vestibular‐related neurons are known for a long time (Baker et al. 1984b; Schor et al. 1985; Kasper et al. 1988; Angelaki 1992a, 1993; Bush et al. 1993; Angelaki and Dickman 2000; Dickman and Angelaki 2002; Yakushin et al. 2006; Chen‐Huang and Peterson 2010). In particular, the canal–otolith VN neurons showed responses more suggestive for otolith‐related input in the low‐frequency range and canal‐related input in the high‐frequency range (Baker et al. 1984b; Kasper et al. 1988). In the earlier studies, it was suggested that the orientation component of response vector to oscillations in the vertical plane did not depend on a stimulus frequency; however, the gain increase and phase changes were observed with increasing stimulus frequencies (Schor et al. 1985; Kasper et al. 1988). It has been argued that if the orientation of response vector remains stable (<10°) at different frequencies of oscillations in 2‐D, the STC response could not appear (Kasper et al. 1988). In some vestibular‐only translation‐sensitive neurons of VN, the direction of maximum sensitivity to translation (i.e., unitary vector) was also frequency dependent (Chen‐Huang and Peterson 2010).

Semicircular canal afferents with regular firing intervals have rotational responses that are linearly related to the angular head velocity, while the canal afferents with an irregular discharge show phase advance and gain enhancement with increasing frequencies of head movements (Fernández and Goldberg 1971; Goldberg and Fernández 1971a,b; Highstein et al. 1987; Goldberg 2000). Similar differences in regularity discharges are seen in otolith afferents that sense linear accelerations (Fernández and Goldberg 1976a,b; Highstein et al. 1987; Goldberg 2000). It has also been demonstrated that the irregular otolith afferents have lower thresholds with higher response variability compared to regular afferents (Yu et al. 2012). Based on this finding it can be assumed that the changes in total response pattern of central neuron are due to different out‐weights of regular/irregular afferent firing activities at different frequencies and amplitudes of vestibular stimuli.

Furthermore, it has been shown that approximately a third of vestibular neurons exhibited complex tuning to the three‐dimensional translations at a single frequency, when maximum translation response vectors lay >20° from either the horizontal or sagittal plane; whereas the maximum translation response vectors of most simple tuning neurons lay within 20° of one of the planes (Chen‐Huang and Peterson 2006). In other studies, the neuronal response of central pure vestibular neurons to otolith stimulations exhibited broadly tuned and narrowly tuned spatial response properties (Bush et al. 1993; Angelaki and Dickman 2000; Dickman and Angelaki 2004). We previously demonstrated that the otolith polarization vectors of central vestibular neurons can change their orientation toward the spatial vertical axis, and the resting firing rates to upright position can also change after prolonged head side‐down orientation (Eron et al. 2008a, 2009).

These findings suggest that the changes of otolith polarization vector of canal–otolith‐sensitive neurons after orientation adaptation re‐gravity is based on the fact that convergent otolith inputs of these neurons are broadly tuned. It is probably because the central otolith‐related cells may be innervated by the different otolith maculae from the same or both labyrinths (Wilson et al. 1978; Uchino et al. 2001, 2005). In a study with anesthetized animals, the broadly tuned response obtained at small angle sinusoidal pitch and roll tilts was found in ~20% of isolated otolith afferent fibers (Dickman et al. 1991). Furthermore, the afferent signals from a single utricular end‐organ may be sufficient to maintain STC properties (Liu et al. 2013; Newlands et al. 2014). The canal‐related convergence to VN neurons from at least two semicircular canals was also previously reported (Kasper et al. 1988; Uchino et al. 2005; Yakushin et al. 2006; Eron et al. 2008b). Accordingly, the VN neurons may receive complex convergent projections from various semicircular canals and the otolith organs (Curthoys and Markham 1971; Baker et al. 1984a; Sato et al. 2000; Zakir et al. 2000; Uchino et al. 2001, 2005; Dickman and Angelaki 2002; Yakushin et al. 2006; Eron et al. 2008b).

The firing rate of neurons with STC response is described as the amplitude of neural modulations plotted versus the stimulus direction relative to the head orientation, and has been modeled as combined dynamic and static activity in the canal–otolith and otolith‐only neurons. Generally, simple models were characterized by a single transfer function shared by two or three canonical axes, while complex models had two or three transfer functions (Angelaki 1992a, 1993; Bush et al. 1993; Kleine et al. 1999; Angelaki and Dickman 2000; Chen‐Huang and Peterson 2006, 2010; Yakushin et al. 2006). In this study, a new proposed model fits the data for different vestibular afferents of both vestibular modalities (i.e., canal and otolith) with different discharges properties (i.e., regular and irregular) not requiring the variation in the system parameters, gains, and time constants, for different frequencies. To determine how many convergent inputs are sufficient for canal–otolith neurons as a function of frequency and orientation, here we have applied the four‐component model, which is able to predict multiple convergence based on data obtained at a few stimulus frequencies as a single set of system parameters.

Neurons with STC responses are also found in the anterior cerebellar vermis (Manzoni et al. 1995; Pompeiano et al. 1997), rostral fastigial nucleus (Buttner et al. 1999; Kleineet al. 1999; Siebold et al. 1999, 2001; Zhou et al. 2001), and the lateral tegmental field (McCall et al. 2013). The presence of neurons with STC behavior in many vestibular‐related brain structures indicates that STC is a fundamental mechanism responsible for the functioning of vestibular‐related reflexes to provide spatial orientation. The functional significance of STC behavior, however, is not fully understood. It is also still unknown whether the STC behavior is a property of a specific class of neurons, or whether any neuron could display STC behavior under appropriate stimulus conditions such as frequency or adapted orientation of polarization vector (Eron et al. 2008a). In this study, we hypothesize that the adaptation of otolith polarization vector could induce the changes in STC response at some frequencies of the vestibular stimuli.

## Material and Methods

The central neuronal activity of vestibular nuclei was investigated in three monkeys (*Macaca fascicularis*). All experimental procedures were conformed to the Guide for the Care and Use of Laboratory Animals and were approved by the Institutional Animal Care and Use Committee of Ichan School of Medicine at Mount Sinai.

### Surgical procedures

The surgical procedures have been previously described in detail (Sirota et al. 1988; Yakushin et al. 2000). Briefly, under general anesthesia, an acrylic head mount was attached to the skull which allowed the animal's head to be held in stereotaxic coordinates painlessly during experiments. In a second surgery, two coils were implanted on the left eye. A perilimbal coil measured horizontal (yaw) and vertical (pitch) eye position (Robinson 1963; Judge et al. 1980). A second coil, placed on top of the eye approximately orthogonal to the perilimbal coil, measured roll (torsional) eye position (Dai et al. 1994).

### Unit recording

Activity of single neurons was extracellularly recorded with varnished tungsten microelectrodes (80 *μ*m, 2–6 MΩ at 1 kHz) (Eron et al. 2007). The microelectrodes were placed into the vestibular nuclei through a stereotaxic plate, installed inside the head mount, 1–2 mm above the skin. This plate had a 10 × 10 grid of 0.61 mm diameter holes at each mm. Microelectrodes were advanced with a lightweight, mechanical microdrive fixed to the head mount. The abducens nucleus was identified first (Smith et al 1972; Scudder and Fuchs 1992).

Unit activity was converted into pulses (BAK Electronics Inc) of standard amplitude (5V) and duration (0.5 msec). Pulses were delayed relative to the action potentials by a fixed time interval of 0.5 msec. The time of spike occurrence was stored relative to the nearest sampling time with the assumption that only one spike could occur within each sampling period (1.0 msec).

Voltages related to eye position and to chair rotation about different axes were amplified and filtered with low‐pass 40 Hz filter and then digitized at 1 kHz/channel with 16‐bit resolution (Data Translation Inc), and stored for off‐line analysis. Position‐related voltages were smoothed and digitally differentiated by computing the slope of the least squares linear fit, corresponding to a filter with a 3 dB cutoff above 40 Hz, the cutoff frequency of the filters used for data acquisition.

### Data collection

Firing characteristics of 55 pure vestibular neurons were studied (Table 1). Seven of them were pure canal‐related neurons and 34 canal–otolith neurons, including vestibular‐only and vestibular‐plus‐saccade neurons. Activities of these types of neurons classify them as head velocity‐related and non‐eye movement‐related neurons; except that vestibular‐plus‐saccade neurons pause in association with saccades in one or more directions (Fuchs and Kimm 1975; Scudder and Fuchs 1992). While there was no evidence to confirm the difference in the activity of vestibular‐only and vestibular‐plus‐saccade neurons with regard to STC behavior, for simplicity, all these neurons below are referred to as canal–otolith neurons. We also recorded from a class of neurons that respond only to head oscillations or static tilts about spatial horizontal axis – 14 otolith‐only neurons. Thus, in this study, we described two classes of pure vestibular cells: canal–otolith and otolith‐only neurons.

**Table 1:** Single or multiple convergent inputs of the tested units

Type of convergence	Number of identified units			
	M0101	M0102	M8552	All animals (%, *n*)
Lateral canal only	1	–	1	4% (2)
Vertical canal only	1	–	3	7% (4)
Lateral canal + vertical canal	‐	1	–	2% (1)
Vertical canal + Otolith	8	2	3	24% (13)
Lateral canal + Otolith	3	3	–	11% (6)
Lateral canals + Vertical canal + Otolith	6	7	2	27% (15)
Otolith‐only	5	3	6	25% (14)
Total	24	16	15	100% (55)

### Histological verification of the recording sites

**Figure 1:**
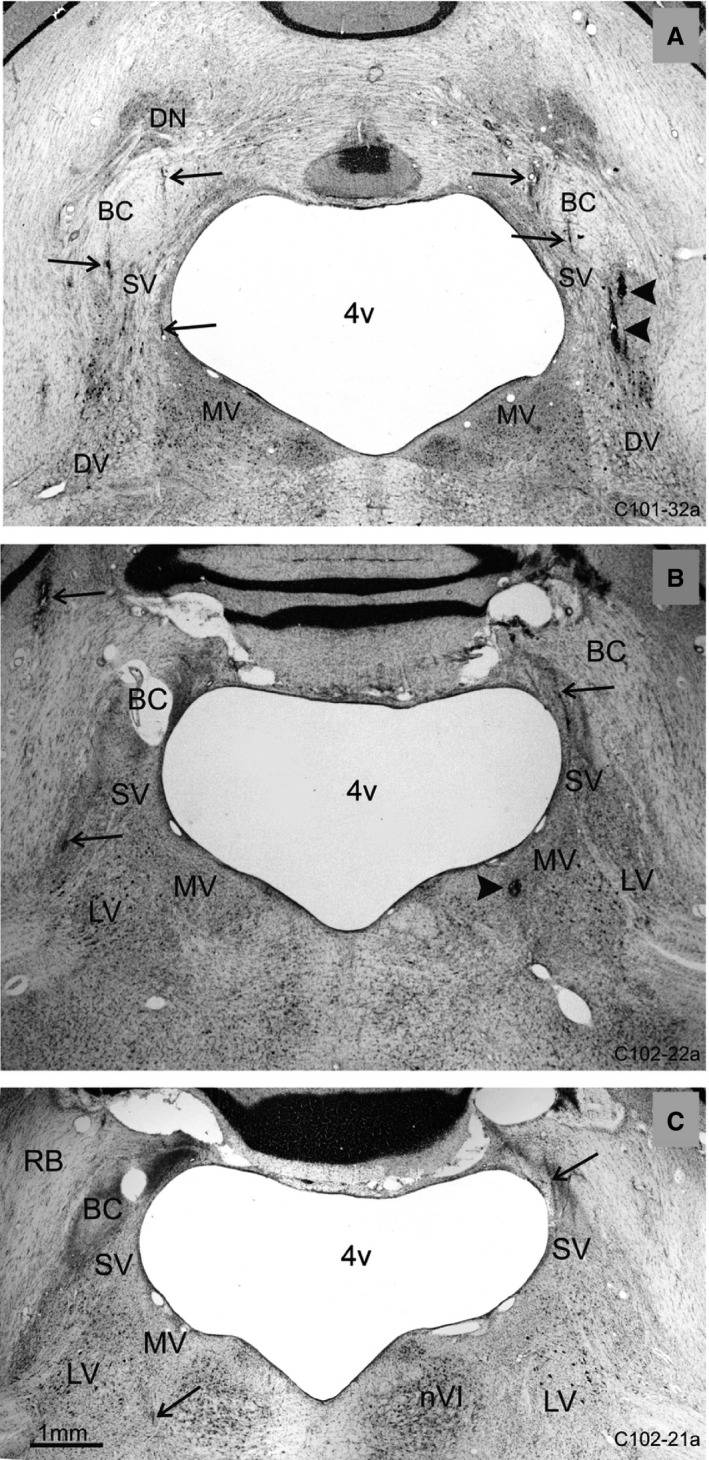
Photomicrographs of three frontal sections through the vestibular nuclei of two monkeys C101‐07 (A) and C102‐07 (B and C), showing some of the glial scars marking the electrode tracks (arrows) which yielded the unit recordings. In (A) C101‐07 the tracks are centered on the SV of both sides, and on the right side the two darker, fresh tracks (containing erythrocytes) mark the site of the DiO injection (arrow head). In (B) C102‐07 the unit recording tracks also pass through rostral SV, and the site of the DiO deposit can be seen in right MV (arrow head); (C) is a more rostral section of C102‐07 than B, and recording tracks are found in the rostral SV of both sides and on the border of the left rostral MV and LV lateral to nVI (arrowhead). Abbreviations are: 4v – fourth ventricle; BC – brachium conjunctivum; DN – dentate nucleus; DV – descending vestibular nucleus; LV – lateral vestibular nucleus; MV – medial vestibular nucleus; nVI – abducens nucleus; RB – restiform body; SV – superior vestibular nucleus.

Histological reconstruction of recording sites in the vestibular nuclei was aided by covering the recording electrode tip with NeuroTracer DiO labeling paste (Molecular Probes, N22881) (DiCarlo et al. 1996). At the termination of experiments, the animals were sacrificed with an overdose of barbiturate and perfused transcardially with 4% paraformaldehyde in 0.1 mol/L phosphate buffer. The brain was removed, blocked in the stereotaxic plane and equilibrated in increasing concentrations of sucrose (10–30%) in 0.1 mol/L phosphate buffer for freeze cutting. Transverse serial sections were cut on a cryostat (Leica) at 40 *μ*m and mounted on gelatinized glass slides. Every sixth section was counterstained with cresyl violet to visualize the gliosis marking the electrode tracks and to determine their location within the vestibular nuclei. In addition, adjacent unstained slides were inspected for the presence of fluorescent electrode tracks, which are labeled by arrow heads in Figure 1.

The location of the recording electrode in two animals is shown in Figure 1. The sites of electrode penetrations were located in the right superior vestibular nucleus (SV) (Fig. 1A, arrowheads). The arrows point to penetrations made by the recording electrodes as they traversed the brain toward recording sites (Fig. 1A–C). In the second animal, many tracks were visible from electrode penetrations in the SV, with the site of DiO deposit in the right medial vestibular nucleus (MV) (arrowhead Fig. 1B). The marked electrode tracks show that majority of pure canal‐related and canal–otolith neurons were recorded in the rostral MV and in SV, while the otolith neurons were recorded more rostrally at the MV/LV border (Fig. 1C, arrow on left side). The histology is not available for the third animal.

Thus, in this study, the canal–otolith neurons were recorded predominantly in SV and MV nuclei, while the otolith‐only neurons were in a rostral part of MV closely to the LV nuclei. The similar locations of the canal–otolith and otolith‐only cells were reported in VN of primates (Scudder and Fuchs 1992; Tomlinson et al. 1996; Angelaki and Dickman 2000; Dickman and Angelaki 2002; Chen‐Huang and Peterson 2006, 2010; Yakushin et al. 2006).

### Coordinate systems

**Figure 2:**
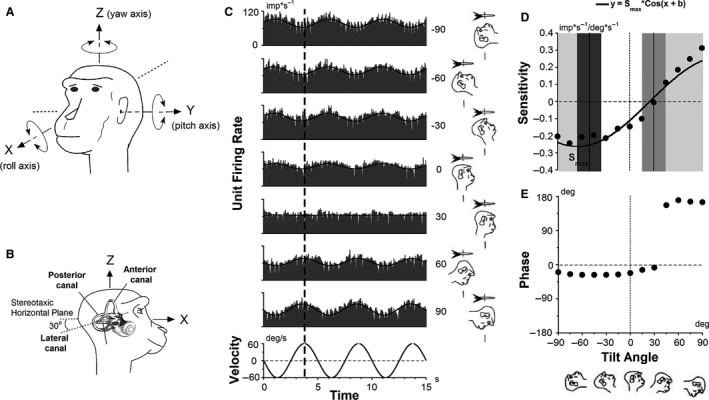
(A) Diagram of spatial coordinate system and stimuli axes in 3‐D. (B) The position of the right labyrinth of a monkey in stereotaxic head coordinate system. (C) Modulation of the firing rate of a vestibular neuron (Unit#4) during rotation about a spatial vertical axis with the head tilted forward and backward in 15° increments. The figure shows FR modulations with 30° increments. (D, E) Temporal sensitivities and phases of the neuron plotted as function of head orientation and fitted by a cosine (dark line in **D**). This central vestibular neuron did not modulate relative to velocity with head tilted at 30° forward, but modulated maximally (0.26 ± 0.017 imp*s^−1^/deg*s^−1^) with head tilt backward at 65 ± 4°. That indicates input from ipsilateral VC; namely, this neuron had input from right posterior VC. Range of maximal spatial sensitivities relative to head orientation in pitch axis for different canal convergences in central neurons: single LC (tilt forward at 30 ± 15°, gray segment), single VC (tilt backward at 50 ± 15°, dark gray segment), LC and VC from different labyrinths (white segment), LC and VC from same labyrinth (light‐gray segments).

Animals were tested in a multi‐axis vestibular stimulator enclosed in a light‐tight cylinder/box. Prior to the start of the experiment, the head was fixed in the stereotaxic horizontal plane when the animal was upright (see diagram in Fig. 2A). The head stimulus coordinate frame was defined by three orthogonal axes: the *X*‐axis (naso‐occipital, positive direction back‐to‐front), *Y*‐axis (interaural, positive from the left ear), and *Z*‐axis (long body axis, positive up).

During identification of semicircular canal convergent inputs the animal's head was sinusoidally rotated about the spatial vertical (yaw) axis at different head orientations in pitch. During determinations of static otolith convergent input or testing STC responses, the animal's head was tilted or sinusoidally rotated, respectively, about the spatial horizontal (pitch) axis at different head positions in yaw plane in darkness (see below).

### Experimental approach

At the first step of this study, the STC properties of central vestibular neurons tested at different frequencies were characterized by the convergence of regular and irregular inputs for otolith‐only neurons or regular canal and regular otolith inputs for canal–otolith neurons using a two‐component model to describe the dynamic and static inputs, respectively. At the second step, the complex model, that assumes regular and irregular canal‐related and regular and irregular otolith‐related inputs, was fit to data for canal–otolith neurons. Then it was estimated whether predicted and determined canal‐ and otolith‐related inputs were correlated for neurons that were adequately fit by two‐component and four‐component models.

The main goal of this study was to determine whether the STC characteristics of vestibular neurons could be altered after orientation adaptation (see below) if the otolith polarization vector is changed. To achieve this goal, the orientation and sensitivity of canal and static otolith inputs before and after orientation adaptation were determined. The STC properties were also tested before and after orientation adaptation. Finally, the orientation adaptation was performed and the changes in RVO (response vector orientation) were determined. The received data were fitted by the models to identify whether the changes in orientation of polarization vector fully explain changes in neuronal STC response obtained after adaptation.

### Determining canal‐related inputs to central neurons

To assign modulation of unit firing rate (FR) to particular semicircular canal activation, the animal was rotated sinusoidally about a spatial vertical (yaw) axis at 0.2 Hz, peak velocity 60°/sec, while the head was upright or tilted up to 90° forward and backward in 15° increments (Fig.**** 2). Modulations of neuronal FR and stimulus velocity were fit by sinusoids at the frequency of stimulation (Fig. 2C). The ratio of amplitudes of FR and stimulus velocity is referred to as the temporal sensitivity (Fig. 2D). The phase difference between the FR and stimulus is referred to as the temporal phase (Fig. 2E). The temporal sensitivities of the unit FR's were plotted as a function of head tilt and fit with a cosine function. The amplitude of the fit is referred to as the spatial sensitivity (gain), while the phase difference of the peak response to the upright position is referred to as the spatial phase (see in detail Eron et al. 2007).

It was previously demonstrated that lateral canals (LC) are maximally activated when the head is tilted forward about 30°, while vertical canals (VC) are activated when the head is tilted 50° backward (Yakushin et al. 1998). Based on variation in the spatial phase of primary vestibular afferents (Fig. 2B; see in detail Reisine et al. 1988), we assumed that central vestibular units received convergent input from a single semicircular canal if the spatial phase of the response did not deviate more than ±15° from the canal plane (Kasper et al. 1988; Yakushin et al. 2005a). For instance, the neuron (Unit #4) shown in Figure 2 had maximal FR modulation with the head tilted ≈60° backward, indicating input from ipsilateral VC; and this neuron did not modulate relative to velocity when the head was tilted at 30° forward, indicating absence of a LC input (Fig. 2D and E).

If a unit had maximal sensitivity when the head was tilted in pitch between −35° and 15°, it indicated that the unit received inputs from both the LC and VC located on opposite sides (Yakushin et al. 2006). Similarly, if the spatial phase was higher than 45° forward and less than −65° backward, there were convergent inputs from the LC and VC on the same side (Eron et al. 2008b). To assign modulation of the neuronal FR to a particular canal, as in our previous studies, we assumed that all inputs were excitatory. Therefore, an increase in the FR comes from the canal that is activated by this rotation.

The oscillation about a spatial vertical axis, however, could only determine whether excitatory vertical canal‐related input comes from the ipsi‐ or contralateral side. Therefore, to determine whether this vertical canal‐related input arose from anterior or posterior canals, the animal's head was also oscillated about a spatial horizontal axis with the head oriented in yaw in 15° increments over 180° at 0.2 Hz (see below). If a unit was maximally modulated by head velocity in the plane of the left anterior VC and right posterior VC, we assumed that the convergent input derives from the canal which was activated during rotation in that direction.

### Identification of static otolith convergent input in central vestibular neurons

**Figure 3:**
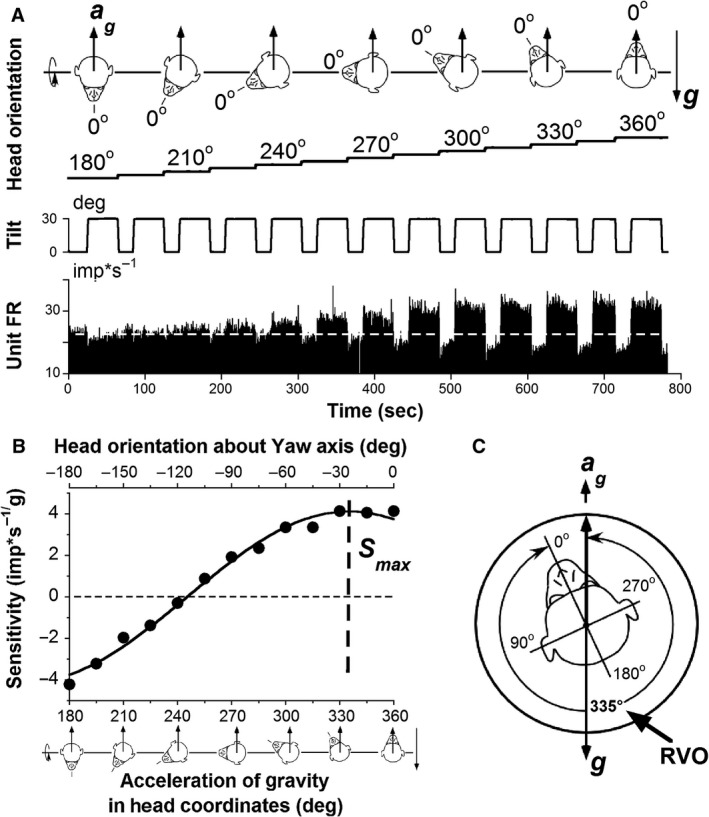
Determination of response vector orientation (RVO) in the central otolith‐related neurons (example of an otolith‐only neuron, Unit #3_o_). (A) Changes in neuronal firing rate (Unit FR) in response to 30° head tilts (Tilt) in various head orientations in yaw with regard to acceleration of gravity *a*_*g*_ in head coordinates. Inset above shows orientation of *a*_*g*_ fixed in space and relative to the head (upward arrows). Values below are the angles for each inset. Resting FR is neuronal spontaneous discharge in upright head position (white dashed line). (B) Unit FR from A, plotted as a function of the angle of *a*_*g*_ in head coordinates (lower x‐scale) and converted to sensitivity. The head orientation in yaw is labeled on the upper x‐scale. The vertical dashed line indicates the location of the peak of the sinusoidal fit through the data (*S*_*max*_). (C) Summary of the RVO computation. The angle corresponding to RVO was 335° in head horizontal plane.

Static otolith input was characterized by the RVO, which is a projection of the polarization vector onto the head horizontal plane (*X*–*Y* plane) (Schor et al. 1984; Eron et al. 2008a, 2009). Because the otolith organs respond to linear acceleration, the orientation of the equivalent acceleration of gravity (*a*_*g*_), whose direction is opposite or 180° from gravity *(g)*, was considered to be the stimulus (Fig. 3A, inset on the top). Thus, when the head was tilted nose‐down, *a*_*g*_ was along the naso‐occipital axis at 180° in head coordinates. Side‐down head tilts to the left or nose‐up tilts correspond to *a*_*g*_ at 270° and 360° in head coordinates, respectively.

To determine the RVO, animals were tilted from upright by 30° or 60° for different head orientations about a yaw axis from 180° (tilt backward) to 360° (tilt forward) in 15° increments (Fig. 3A, top panel). Tilt stimulus can produce an initial increase in FR (Fig. 3A, bottom panel) due to activation of the semicircular canal‐related and/or dynamic otolith‐related inputs to the neuron, which declined to a steady state level with a time constant of less than 20 sec. Therefore, while the head remained tilted in each position for ≈40 sec, only the last 20 sec were analyzed. This method significantly reduced the contribution of the dynamic otolith input for the majority of neurons that were tested. Unit FR's were plotted as a function of the direction of *a*_*g*_ in yaw head plane (Fig. 3B) and converted into sensitivities (imp*s^−1^/g, (Schor et al. 1984)). Sensitivity curves were then fit with a sinusoid, *y* = *S*_max_*cos(x+*β*), to determine the maximal sensitivity (spatial gains, *S*_max_) and the head orientation in yaw at which this maximal sensitivity occurred (*β*, spatial phase) (Fig. 3B), i.e., the RVO (Fig. 3C).

### Identification and criteria of STC properties

**Figure 4:**
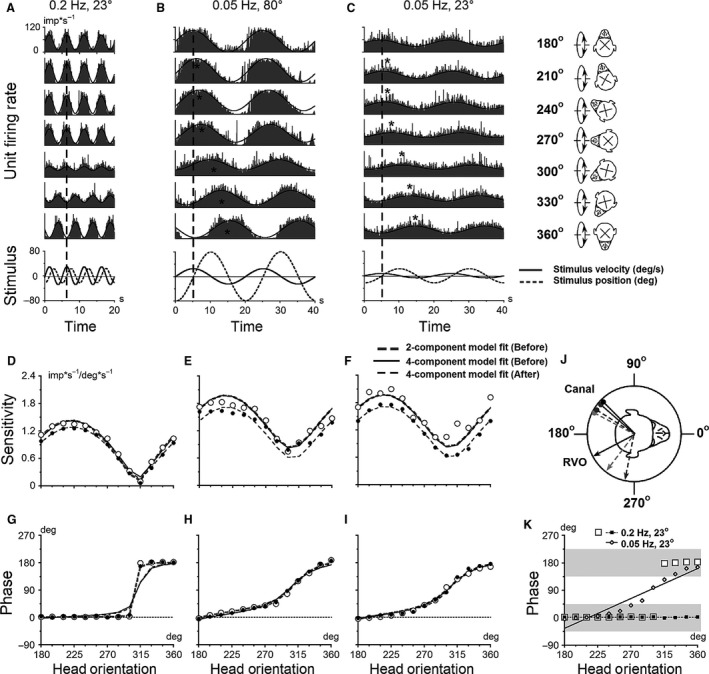
Responses of a canal–otolith convergent neuron (Unit #5) during sinusoidal rotations about an earth‐horizontal (pitch/roll) axis with different head orientations in yaw plane. (A) Modulation of unit FR for oscillations at 0.2 Hz with peak tilt amplitude of 23°. (B, C) Modulations of unit FR for oscillations at 0.05 Hz with peak tilt amplitude of 80° and 23°, respectively. Each unit was tested at 15° increments in yaw axis. The figure shows FR modulations with 30° increments for oscillations before orientation adaptation re‐gravity. Inset on the right is an angle cartoon of relative head orientation in yaw to the axis of oscillations. Stimulus velocity (solid line) and stimulus position (dotted line) during oscillations at different tested frequencies and peak tilt amplitudes are shown on the bottom traces. Bold curves in each panel represent the sinusoidal fits of the data (A–C). The vertical dashed line indicates a time of the head peak velocity, the asterisks the peaks of the neuronal responses, which varied with the head orientation in yaw plane. Sensitivity (D, E, F) and phase (G, H, I) of the neuron are calculated with respect to velocity and plotted as a head orientation in yaw plane to the axis of oscillation before (A, B, C, open symbols) and after (filled symbols) head re‐orientation for 2 h. The changes in temporal sensitivity and phase as a function of head orientation in yaw were well approximated using the two‐component model (*Eg. 1*) comprising regular canal and otolith inputs (bold dashed gray curves in D–I, data before). The data were also fitted by the four‐component or *complex* model (*Eq.* 4) before (solid black curves) and after (dashed black curves) orientation adaptation re‐gravity. (J) Summary polar plot that shows orientation of RVO (arrows) and canal‐related input (drumsticks) experimentally determined (black) and the two‐component model predicted for 0.2 Hz (solid gray) and for oscillations at 0.05 Hz with peak amplitude of 80° (dashed gray) and 23° (dashed light gray). (K) Calculation of slopes (*α*) using phase changes versus head orientations in yaw for non‐STC (filled squares) and typical STC (open diamonds) responses. Open squares show phase changes of non‐STC behavior for unit modulation at 0.2 Hz with peak amplitude of 23° (A), where unit modulates only to velocity having two levels of phases, ±180° (open squares) thereby the phases can be converted to similar level (filled squares). Open diamonds show phase changes of STC response for the unit modulation at 0.05 Hz with peak amplitude of 23° (C), where phases monotonically change in yaw plane from in‐phase with head velocity to head position and cannot be converted to same level. Each phase curve plotted vs. head orientation was approximated by linear function: *y* = A+ α*x, where *α* – slope of linear function was examined. For non‐STC responses the slope of phase curve was 0.0015 (dotted line) and for STC responses the slope was 1.09 (solid line). The range of temporal phases within ±45° relative to velocity stimulus is shown in gray segments, those for position stimulus is shown in white segments.

To initiate a mixed stimulation of vertical semicircular canals and the otolith organs the animal's head was sinusoidal rotated about the earth‐horizontal (pitch/roll) axis at 0.2 Hz with a peak amplitude of 23° (Fig. 4A) and at 0.05 Hz with peak amplitudes from 23 to 80° (Fig. 4B and C). During the oscillations, the head was orientated at different positions about a yaw axis from 180° to 360° in increments of 15°: the nose‐down/nose‐up rotations; rotations in plane right anterior/left posterior vertical canals; right/left side or roll head rotations; rotations in plane right posterior/left anterior vertical canals; and nose‐up/nose‐down head rotations (see Fig. 4 cartoon on the right). During sinusoidal rotations about the earth‐horizontal axis changes in angular acceleration and velocity activate the vertical semicircular canals, while changes in *a*_*g*_ activate the otolith organs. Thereby, oscillations at different frequencies and with different amplitudes of oscillations activate canal and otolith convergent inputs to different magnitudes and, therefore, affect the total neuronal response. The oscillations at two frequencies (0.2 Hz and 0.05 Hz) with several peak amplitudes of tilts were utilized in this study. During oscillations at 0.2 Hz with 23° amplitude the peak velocity was ~28°/sec and peak acceleration was ~35°/sec^2^ in spatial quadrature, while for stimulation at 0.05 Hz with 80° amplitude the peak velocity and peak acceleration were ~25°/sec and ~8°/sec^2^, respectively (see bottom traces in Fig. 4A and B). Stimuli consisted of sinusoidal tilts at frequency of 0.05 Hz with amplitude of 23°, the peak velocity and peak acceleration were reduced to ~7°/sec and ~2°/sec^2^ (Fig. 4C, bottom trace). Note, in a few neurons, the head was oscillated at 0.05 Hz with peak amplitudes of tilts 50° and 60° (Unit #4, 11, and # 13, 16 in Table 2). Those data for peak tilt amplitudes within 50–80° for head sinusoidal tilts at 0.05 Hz were combined.

**Table 2:** Orientations of vertical canal and otolith inputs experimentally measured and predicted by two‐ and four‐component models before and after orientation adaptation

Unit#	Before RVO adaptation						After RVO adaptation					
	Experimental measurements		Model prediction				Experimental measurements		Model prediction			
	VC	RVO	RC	IC	RO	IO	VC	RVO	RC	IC	RO	IO
1	315	120	315	278	127	283	315	88	315	257	81	256
2	135	292	160	‐	303	125	135	271	160	‐	313	7
3	45	180	44	7	185	208	45	179	45	29	178	222
4	225	66	213	‐	38	195	225	103	210	‐	55	192
5	135	208	133	176	259	‐	135	x	133	175	265	‐
6	225	25	232	‐	19	180	225	22	228	‐	−1	153
7	225	57	230	NA	13	NA	225	90	234	NA	108	NA
8	45	243	51	NA	273	NA	45	270	48	NA	272	NA
9	45	271	83	NA	227	NA	45	255	62	NA	211	NA
10	45	242	65	‐	242	143						
11	225	30	222	258	63	‐						
12	135	x	132	‐	275	117						
13	315	x	336	‐	133	258						
14	45	101	42	‐	123	50						
15	315	91	298	‐	104	349						
16	225	43	232	‐	44	190						

Hyphen (‐) indicates that the significant input was not identified by four‐component model. The “x” indicates that RVO was not experimentally measured. The “NA” indicates that unit was tested at only at 0.2 Hz; and only two‐component model predictions of regular canal and regular otolith inputs are shown for Units #‐7‐9.

We assumed that the modulation was in‐phase with stimulus velocity if the temporal phases were larger than ±45° from stimulus position (Fig. 4K, gray segments), otherwise we assumed that the modulation was in‐phase with stimulus position (Fig. 4K, white segments). Units with non‐STC responses had sensitivity change as a function of head orientation, while temporary phase remain the same. There was a head orientation at which the sensitivity was zero and temporal phase change by ±180 after passing zero orientation (Fig. 4K, filled squares). Units with STC responses, had the temporal phase changes monotonically with yaw head orientation, while sensitivity remained above 0 (Fig. 4K, open diamonds). A slope for the phase versus head orientation curve, *α*, was then computed. We assumed that units had STC characteristics when the temporal phase of the unit's FR changed ≥45° when head orientation in yaw was altered over 180°. This corresponds to a slope of 0.5 ≤ *α*<1.0. In this study, we assumed that a unit had nonzero sensitivity if it remained ≥20% of maximal value in all head orientations in yaw, and a slope was <0.5. All units in the study were tested with this criterion.

Figure 4 shows the response of a canal–otolith neuron (Unit#5) with typical STC properties that received input from left posterior canal at 135° (Fig. 4J, black drumstick). The RVO of the otolith input was at 208° (Fig. 4J, black arrow). The angle of the difference between vertical canal input and the RVO was 73°. At 0.2 Hz, the neuron was modulated only in‐ or out‐of‐phase with head velocity (Fig. 4A, dashed line). Jump of temporal phase occurs at a head orientation at ≈ 315° where the sensitivity is close to zero (Fig. 4D, G; *α *= 0.0015, *R* ^2^=0.004, *P* = 0.83 for non‐STC example). Since both canal‐ and otolith‐related inputs were activated at 0.05 Hz, this unit was modulated out‐of‐phase with head velocity when the head was oriented in yaw at 225°, stimulating the left PC (Fig. 4B, C, dashed lines and asterisks). The FR was modulated out‐of‐phase with head position when the head was oriented in yaw about 300° (Fig. 4B and C, asterisks). The unit temporal sensitivity was above 0.6 imp*s^−1^/deg*s^−1^ in all head orientations (Fig. 4E, F) and the temporal phases gradually changed from being in‐phase with head velocity to head position as the head orientation in yaw was altered during testing (*α* = 1.07 and 1.09, *R* ^2^=0.887 and 0.925, *P* < 0.001; Fig. 4H, I for STC examples with peak tilt amplitudes 80° and 23°). Thus, the activity of this neuron was consistent with the hypothesis that the STC properties are the result of a «summation» of a vertical canal and static otolith inputs for both peak amplitudes of sinusoidal rotations at 0.05 Hz.

### Model‐based analysis of STC properties

The temporal sensitivities and phases of canal–otolith neurons with STC characteristics obtained at a single frequency of the head oscillation could be well fit by a model that assumed only regular canal and regular otolith inputs (Yakushin et al. 2006). It was not clear, however, whether the model would fit the data obtained at several frequencies, peak velocities and/or amplitudes of the head oscillation.

The four‐component model was implemented in custom C++ program with MS Excel interface in which each of the four inputs could be fixed or allowed to vary. The fits were obtained by representing the temporal gains and phases as vectors in the complex plane. A multiple linear regression (MLR) algorithm was used to obtain the best model prediction values in the least mean squares approach. The model fits of data in the complex plane were then converted back to gain and phases, and plotted over experimental data. When only regular canal and otolith inputs were assumed (two‐component model), the irregular canal and irregular otolith inputs were fixed at zero value. Similarly, when data obtained from otolith‐only neurons were analyzed, the sensitivities of regular and irregular canal inputs were fixed at zero.

The model‐based analysis was accomplished using the following steps:

We first fit the data with the model that assumed dynamic and static vestibular inputs, especially only regular semicircular canal and otolith inputs to the canal–otolith neuron (two‐component model):

(1) $$\begin{array}{cc} \text{NR}\left(\theta ,\omega \right)=\;\; & AH_{\mathrm{RC}}\left(\omega \right)\text{Cos}\left(\theta +\varphi_{\mathrm{RC}})\right)+\\ & BH_{\mathrm{RO}}\left(\omega \right)\text{Cos}\left(\theta +\varphi_{\mathrm{RO}}\right), \end{array}$$

where *ω* is the radian frequency of stimulus oscillation, *θ* is the angle of head yaw orientation, NR is a neural response which is a function of *θ* and *ω*. *A* and *B* are constant gains for canal and otolith components, *φ*_RC_, *φ*_RO_ – phase shifts of canal and otolith components, H_RC_ *(ω)*, H_RO_ *(ω)* are transfer functions as a function of radian frequency of regular canal (RC) and regular otolith (RO) in response to angular head velocity inputs.

The system transfer functions H_RC_ *(ω)* and H_R_ *(ω)* were chosen as follows:

(2) $$H_{\mathrm{RC}}\left(\omega \right)=30*s/\left(1+30*s\right)$$

(3) $$H_{\mathrm{RO}}\left(\omega \right)=1/s$$

where *s* is complex variable in Laplace form. For this application *s* = *jω*.

To identify the irregular canal (IC) and irregular otolith (IO) convergent inputs to the canal–otolith neurons these data were fit using a four‐component model:

(4) $$\begin{array}{cc} \text{NR}\left(\theta ,\omega \right)=\;\; & AH_{\mathrm{RC}}\left(\omega \right)\text{Cos}\left(\theta +\varphi_{\mathrm{RC}}\right)+\\ & BH_{\mathrm{RO}}\left(\omega \right)\text{Cos}\left(\theta +\varphi_{\mathrm{RO}}\right)+\\ & CH_{\mathrm{IC}}\left(\omega \right)\text{Cos}\left(\theta +\varphi_{\mathrm{IC}}\right)+\\ & DH_{\mathrm{IO}}\left(\omega \right)\text{Cos}\left(\theta +\varphi_{\mathrm{IO}}\right) \end{array}$$

where IC refers to irregular canal and IO to irregular otolith inputs. *C* and *D* are constant gains for irregular canal and irregular otolith components, *φ*_*IC*_ and *φ*_*IO*_ are a phase shifts of irregular canal and irregular otolith components. H_IC_ *(ω)* and H_IO_ *(ω)* are a frequency dependence of irregular canal and irregular otolith afferents re head velocity (Goldberg and Fernández 1971a; Fernández and Goldberg 1976a).

Note, that the data of otolith‐only neurons were also fitted by the two‐component model to determine the spatial sensitivity and phase for regular and irregular otolith inputs, respectively:

(5) $$\begin{array}{cc} \text{NR}\left(\theta ,\omega \right)=\;\; & BH_{\mathrm{RO}}\left(\omega \right)\text{Cos}\left(\theta +\varphi_{\mathrm{RO}}\right)+\\ & DH_{\mathrm{IO}}\left(\omega \right)\text{Cos}\left(\theta +\varphi_{\mathrm{IO}}\right) \end{array}$$

Regardless of which model was applied, we assumed that a convergent input was significant if its sensitivity was more than 0.05 imp*s^−1^/deg*s^−1^. This is somewhat smaller than the value determined experimentally by others (McArthur et al. 2011). However, this value was statistically significant (*P* < 0.05) in our data model fit.

### Orientation adaptation

To induce orientation adaptation, the animal remained tilted 90° left or right side down and was held in this orientation for two hours (Eron et al. 2008a). Together with the pre‐ and post‐testing for STC characteristics, each neuron had to be recorded for at least six hours. Therefore, to ensure that the recordings maintained stable from the same neuron throughout the recording session, neuronal FR was continuously monitored and canal‐ and otolith‐related inputs were determined before and after orientation adaptation (Eron et al. 2007). We adapted every neuron that we recorded, but because of the technically demanding protocol, the quality of neuronal recording remained stable for only nine canal–otolith neurons and five otolith‐only neurons.

As we previously demonstrated, there were substantial changes in RVO of the majority of canal–otolith neurons, while changes in RVO of otolith‐only neurons were much smaller (Eron et al. 2008a, 2009). Orientation adaptation was used to induce changes in orientation of the otolith‐related input of VN neurons and to determine how it would affect STC behavior and whether such changes could be accounted for by our model.

The STC properties were tested with a small set of frequencies before and after orientation adaptation re‐gravity since a full set of tests required a significant amount of time and was limited by the duration of experiment per day. All canal and otolith identification tests and STC tests were performed before and after orientation adaptation for 2 h. The lack of any changes in the spatial orientation of canal‐related inputs and types of the other convergent inputs to a cell during an experiment confirmed that the same neuron was recorded throughout (Eron et al. 2007).

### Statistical analysis

The significance of the sinusoidal fits through the data during determination of RVO and difference of RVO before and after spatial adaptation were estimated using an *F*‐statistic (*P* < 0.05).

For STC model‐data comparison, the temporal gains and phases at each head orientation about the yaw axis were converted to points on the complex plane. A scalar function was constructed as a sum of distances between experimental points and corresponding points from the model. The function was minimized by varying fit parameters using the gradient descent method. The quality of the model fits were estimated by coefficient of determination R^2^ and considered statistically significant for *P* < 0.05. Differences between the predicted model curves before and after orientation adaptation were also determined by an *F*‐statistic.

The model‐predicted spatial orientations of the convergence for regular canal and regular otolith inputs in yaw were plotted against experimentally estimated values for these inputs, and the data were fit by a linear regression model. The significance of the linear regression was estimated based on the critical value of the coefficient of determination *R* ^2^ (*P* < 0.05) (Glantz and Slinker 1990). The goodness of the model predictions was characterized by the difference of the slope of the linear regression from unity and the deviation of experimental data from a linear regression line.

In order to obtain a robust statistic for the small data set of neurons, the data summary was presented as median Q_2_ with the lower and upper quartile values (*Q*_1_ : *Q*_3_). To compare data “before” and “after” adaptation, the Wilcoxon *T* test was used as nonparametric alternative to the Student's *t*‐test.

## Results

### Determining convergent vestibular inputs to neurons

Convergent vestibular inputs were verified in 41 canal–otolith and 14 otolith‐only neurons. STC characteristics were determined for 26 canal–otolith and seven otolith‐only neurons before adaptation and for nine canal–otolith and five otolith‐only neurons after orientation adaptation.

Eight canal–otolith neurons had lateral canal and no vertical canal input. The spatial phase of these units was 25° (15.93 : 35.53°) and spatial sensitivity was 0.69 imp*s^−1^/deg*s^−1^ (0.38 : 0.88 imp*s^−1^/deg*s^−1^). Fifteen had vertical canal and no lateral canal input. Their spatial phase was −64° (−64.99 : −48.68°) and spatial sensitivity was 0.61 imp*s^−1^/deg*s^−1^ (0.33 : 0.68 imp*s^−1^/deg*s^−1^). The remaining neurons received convergent input from both lateral and vertical canals. Seven neurons had spatial phases less than 15° and greater that −35° (median = −19°, −28.34 : −9.05°), indicating convergent inputs from both lateral and vertical canals located on opposite sides of the brainstem (Yakushin et al. 2006; Eron et al. 2008b). Their average spatial sensitivity was 0.5 imp*s^−1^/deg*s^−1^ (0.30 : 0.68 imp*s^−1^/deg*s^−1^). The spatial phases of nine other neurons were greater than 45° or less than −65°, indicating convergent inputs from the lateral and vertical canals on the same side (Eron et al. 2008b). The median spatial sensitivity for these nine units was 0.60 imp*s^−1^/deg*s^−1^ (0.32 : 1.01 imp*s^−1^/deg*s^−1^). Thus, 39% of the canal‐related neurons had convergent input from at least two canals, located on the same or opposite sides of the head. Most of the canal‐related neurons (83%) also received input from the otolith organs, while few neurons (15%) received input from only one canal without otolith input (Table**** 1). The resting FR in upright position was 36.07 (23.85 : 51.49 imp/s) in canal–otolith neurons and 41.57 imp/s (25.98 : 76.0 imp/s) in otolith‐only neurons. Thus, the resting FR was similar in both groups of central cells (*P* = 0.245).

Neither canal–otolith neurons nor otolith‐only neurons had specific orientation of their otolith polarization vectors. The sensitivity to head tilt was 7.59 imp*s^−1^/g (5.75 : 15.12) in canal–otolith neurons and 15.36 imp*s^−1^/g (9.49 : 23.46) in otolith‐only neurons, indicating that sensitivity to otolith stimulation was mainly smaller for canal–otolith for otolith‐only neurons (*P* = 0.017).

### Two‐Component Model Predictions

#### Prediction of regular canal and regular otolith inputs in canal–otolith neurons

Twenty‐six canal–otolith neurons were tested by oscillation about spatial horizontal axes with different head orientations in yaw. Eleven of 26 tested units showed STC properties (42%) when the head was oscillated about a spatial horizontal axis at 0.2 Hz. There was a further attempt to fit the data obtained from 26 units tested at 0.2 Hz with this two‐component model. The responses of eight neurons (31%) failed to be an adequate comparison; while in three of them RVO was not tested (Table 2) and in five of them neurons modulated at 0.2 Hz close to being in‐ or out‐of‐phase with head velocity in all head orientations in yaw, and the two‐component model did not identify significant regular otolith‐related input for this frequency of oscillation. For the remaining 18 neurons (69%), the simple model accurately fit experimental data.

When orientation of the vertical canal input was plotted versus the model‐predicted orientation for 18 tested neurons (Figure S1A), the slope of the linear fit was close to unity (0.97, *R* ^2^=0.964, *P* < 0.001). There was also a significant correlation between the measured and predicted RVOs, and the slope of the linear regressions was 0.66 (*R* ^2^=0.643, *n* = 18, *P* < 0.001; Figure S1B). This demonstrates that two‐component model was capable of successfully predicting the neuronal behavior of many but not all canal–otolith convergent units. This also indicates that some neurons may have more complex convergent input.

Thirteen of the 26 neurons tested at 0.2 were recorded long enough and were also tested at 0.05 Hz: eight of 13 neurons were tested with peak amplitude above 50°, and eight neurons were tested with peak amplitude 23°, and two neurons were tested at both stimulus conditions. The experimental data and values predicted by the two‐component model were compared (Figure S1C–F). Eleven of 13 neurons (85%) showed STC properties at 0.05 Hz. The data for peak tilt amplitudes of head oscillations above 50° are shown in Figure S1C, D. The slope of the linear regression for the canal input was 0.94 (Figure S1C, *R* ^2^=0.958, *n* = 8, *P* < 0.001). The RVO in two of these eight neurons was not determined; thereby the comparison was performed for six remaining neurons. Thus, for static otolith input, the slope was 1.01 (Figure S1D, *R* ^2^=0.921, *n* = 6, *P* < 0.001). When the head was oscillated with peak amplitude of 23°, the slope of the linear regression was 0.89 for the canal input (Figure S1E, *R* ^2^ = 0.931, *n* = 8, *P* < 0.001), and was 1.03 for otolith inputs (Figure S1F, *R* ^2^ = 0.954, *n* = 8, *P* < 0.001). This indicates that the two‐component model could significantly fit the data obtained at each frequency individually if parameters of fit for the same neuron were allowed to vary between the frequencies. In some neurons predicted orientations of both regular canal and regular otolith inputs were similar for 0.2 and 0.05 Hz frequencies (Units #3, 10, 11, 14, 16). Other neurons had differences in the predicted orientation of the regular otolith input for oscillations at 0.2 Hz (Figure S1B) and regular canal input for oscillations at 0.05 Hz with peak amplitude of 23° (Figure S1E). This indicates that these neurons receive additional convergent inputs that are not accounted by the two‐component model. These data further indicate that whether a neuron receives only two convergent inputs cannot be accurately determined from data obtained at a single frequency. Below we provide three specific examples of fitting the data obtained at two frequencies with the two‐component model.

The first example is shown in Figure 4. This unit received convergent input from the left posterior canal at 135° and had otolith convergent input with a RVO at 208° (Fig.**** 4 inset, black drumstick and arrow). This neuron had STC properties only at 0.05 Hz. This indicates that canal‐related input at 139° dominates in the overall response at 0.2 Hz. The predicted canal‐related input for the data obtained at 0.05 Hz was 148° and 152° for peak amplitudes 80° and 23°, respectively (Fig.**** 4J, gray and light‐gray dashed drumsticks). The difference between experimentally determined and predicted canal‐related input was 4° at 0.2 Hz and 13° and 17° at 0.05 Hz for both peak amplitudes 80° and 23°. The otolith input of this neuron was not identified by the two‐component model at 0.2 Hz frequency, while it was predicted by the model at 0.05 Hz of stimulations. For this frequency, the orientation of otolith inputs was 259° and 233° for peak amplitudes 80° and 23°, respectively (Fig. 4J, gray and light‐gray dashed arrows). Thus, while datasets obtained at each frequency could be well fit by the two‐component model, all data sets could not be fit with a single set of parameters (Fig. 4D–I, bold dashed gray curves), suggesting that additional dynamic components are required.

**Figure 5:**
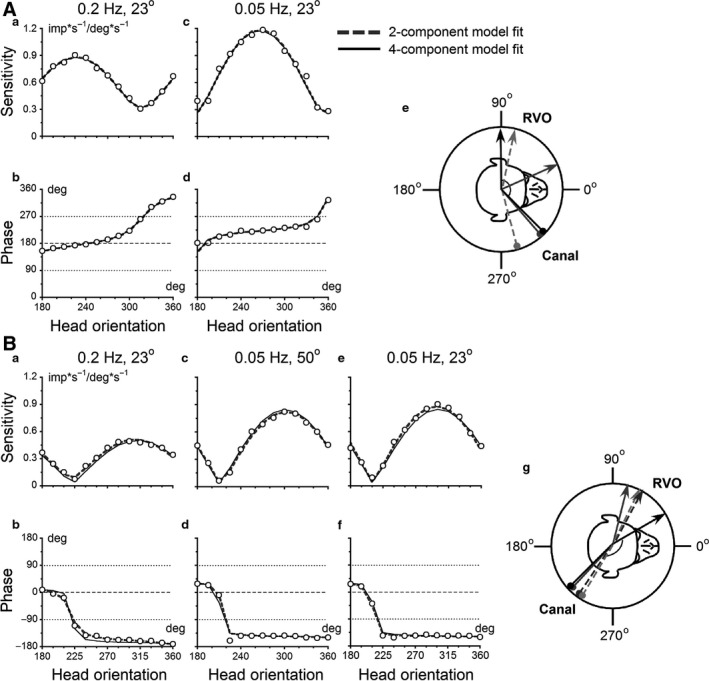
(A, B) Sensitivities and phases obtained from canal–otolith convergent neurons (Unit #15 in A a–d and Unit #11 in B a–f) by oscillations about earth‐horizontal axis at 0.2 Hz and 0.05 Hz in different head orientations in yaw plane relative to the axis of oscillation (abscissa). Open symbols are experimental data; gray dashed curve is two‐component model fits through the data, while solid curve is four‐component model fit. Ae, Bg, Insets show orientations of the semicircular canal (drumsticks) and RVO (arrows) that were experimentally measured (black) and predicted by the two‐component model for 0.2 Hz (solid gray) and for oscillations at 0.05 Hz with peak amplitude of 50° (dashed gray) and 23° (dashed light gray), respectively.

A second neuron (Unit #15) received input from the right anterior canal at 315° (Fig. 5A e, black drumstick) and its RVO was 91° (Fig. 5A e, black arrow). The temporal phase gradually changed from being in‐phase with head velocity to head position as the head was reoriented in yaw (*α* = 1.01, *R* ^2^ = 0.878, *P* < 0.01; Fig. 5A b). Thus, this neuron had STC properties at 0.2 Hz (Fig. 5A a, b). When the same cell was tested at 0.05 Hz, however, the unit did not show clear STC characteristics and the temporal phase was close to being to head position in all tested orientations (*α *= 0.54, *R* ^2^ = 0.760, *P* < 0.01; Fig. 5A c, d). This unit had STC characteristics only at a higher frequency (0.2 Hz), suggesting that the canal‐related activity, which has a phase close to head velocity, had a larger effect at this frequency. At a lower frequency (0.05 Hz), the phase is closer to head position, indicating a dominant otolith convergent input. The canal and otolith inputs predicted by the model at 0.2 Hz were 311° and 24° (Fig. 5A e, solid gray drumstick and arrow), while at 0.05 Hz; they were 285° and 78°, respectively (Fig. 5A e, dashed gray drumstick and arrow). At a lower frequency, the model predicted that the orientation of the canal‐ and otolith‐related inputs would lie close to a single plane (compare dashed gray drumstick and arrow, Fig. 5A e). Consistent with the lack of separation of the canal and otolith inputs, the unit had no STC characteristics at 0.05 Hz. This finding illustrates that the two‐component model could predict the canal‐related input for a high frequency of head oscillation, when the relative contribution of the vertical canal‐related inputs to the total response was stronger, and for the otolith‐related input at a low frequency of oscillation, when the relative contribution of static otolith input was larger.

A third example shows the canal–otolith neurons (Unit #11) with convergent inputs from the right posterior VC at 225° and otolith input at 30° (Fig. 5B e, solid black drumstick and arrow). The angle between the canal and otolith inputs was 165°. Thus, the inputs lie almost in the same plane, and the unit did not show typical STC responses at either frequency tested. During oscillation at 0.2 Hz, the maximal sensitivity was 0.51 imp*s^−1^/deg*s^−1^ at ≈315° (Fig. 5B a), similar to the sensitivity determined during the canal identification test (0.63 imp*s^−1^/deg*s^−1^). This indicates that at 0.2 Hz this neuron predominantly responded to vertical canal activation. The temporal phase of the response was in‐ or out‐of‐phase with stimulus velocity as the head changed its orientation (*α* = 0.011, *R* ^2^=0.001, *P* = 0.919; Fig. 5B b). The model‐predicted orientations of the canal and otolith inputs were 227° and 76°, respectively, when tested at 0.2 Hz (Fig. 5B g, solid gray drumstick and arrow). When the head was oscillated at 0.05 Hz (Fig. 5B c–f), the sensitivity of this unit increased to ≈ 0.85 imp*s^−1^/deg*s^−1^ and its temporal phases shifted toward being in‐phase or out‐of‐phase with stimulus position (*α* = 0.086 and 0.029, *R* ^2^=0.162 and 0.184 for peak amplitudes 50° and 23°, *P* > 0.144). This indicates that the relative contribution of the otolith input increased at this frequency of oscillation. The orientation of the predicted canal input at 0.05 Hz was 236° and 239° for oscillations at 50° and 23°, respectively (Fig. 5B g, gray and light‐gray dashed drumsticks), while orientation of the predicted static otolith input was 62° and 64° for two peak amplitudes (gray and light‐gray dashed arrows). These data suggest that oppositely directed canal and otolith inputs do affect the spatial sensitivity as a result of an interaction of the otolith and canal inputs, but they do not induce typical STC properties at any tested frequency. This shows that even when significant, the static otolith input cannot be determined by oscillation about a spatial horizontal axis if the canal‐related input is dominant.

**Figure 6:**
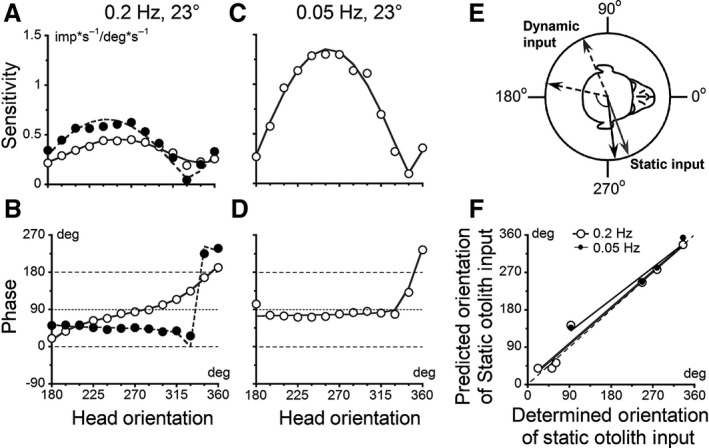
Sensitivities (A, C) and phases (B, D) obtained from an otolith‐only neuron (Unit #1_o_) by oscillations about earth‐horizontal axis at 0.2 Hz and 0.05 Hz with peak tilt amplitude of 23° in different head orientations in yaw plane (abscissa). Symbols are experimental data obtained before (open circles) and after (filled circles) orientation adaptation; curves are two‐component model fits through the data. (E) Orientation of the dynamic/irregular otolith input (dashed arrows) and RVO (solid arrows) that were model predicted before (black) and after (gray) orientation adaptation. (F) Correlation of experimentally determined (abscissa) and two‐component model‐predicted (ordinate) orientation of static/regular otolith inputs is shown for all otolith‐only neurons tested at 0.2 Hz (open circles) and at 0.05 Hz (filled circles).

Thus, the two‐component model accurately predicted the orientation of the canal‐related input for any tested frequencies and amplitudes of head oscillation with a slope of regression line above 0.89, but quality of prediction was better for 0.2 Hz (Figure S1A, C, E). Additionally, the static otolith input was accurately predicted for data obtained at a low frequency of head oscillation, when the static otolith input dominated the canal input (Figure S1D, F). The deviation of the slope of the linear regression from unity and the variation in the data about the slope for static otolith input at 0.2 Hz, indicating that other factors besides static otolith input, such as dynamic otolith sensitivity, could affect the predicted values of RVO (Fig. 6A, B).

#### Prediction of regular and irregular otolith inputs in otolith‐only neurons

STC characteristics were determined for seven otolith‐only neurons when the head was oscillated at 0.2 Hz with peak amplitude 23°. Three of seven neurons showed STC response at this frequency (Table**** 2, Units #1_o_‐3_o_). Experimentally determined RVOs were compared to the static otolith input predicted by the two‐component model (Fig. 6, open symbols). Four of these neurons were also tested at 0.05 Hz. None had STC properties at this frequency, including units that had STC properties at 0.2 Hz.

Although dynamic otolith input was not experimentally determined in this study, the shift of temporal phase at a certain head orientation from being in‐phase with head position at 0.05 Hz toward being in‐phase with head velocity at 0.2 Hz could be explained by the dynamic otolith input.

When an otolith‐only neuron (Unit #1_o_) shown in Figure 6 (open symbols) was tested at 0.2 Hz, it modulated maximally in‐phase with head position for head orientation between 255° and 285° in yaw, while there was modulation in‐phase with velocity when the head was oriented close to 180° and 360° (Fig. 6A and B, open symbols). STC properties of this otolith‐only neuron observed at 0.2 Hz (*α* = 0.82, *R* ^2^=0.938, *P* < 0.001) are similar to that of the canal–otolith convergent neuron shown in Figure 5A. This indicates that additional dynamic (irregular) otolith input oriented almost orthogonal to experimentally determined static (regular) input is the most likely cause of the observed STC behavior (Table 3). During oscillation at 0.05 Hz, this unit did not show STC characteristics and was modulated only in‐phase with head position (*α* = 0.02, *R* ^2^ = 0.006, *P* = 0.809), indicating that dynamic otolith input was not significantly activated at this frequency (Fig. 6C and D). Thus, some otolith‐only neurons could have STC properties at 0.2 Hz frequencies at which both the dynamic and static otolith inputs were activated. The orthogonal static and dynamic otolith inputs were also predicted for two other otolith‐only neurons (Units #2_o_ & #3_o_) with STC responses (Table 3).

**Table 3:** Orientation and sensitivity of the dynamic and static inputs in otolith‐only neurons predicted two‐component model

Unit#	RVO	Two‐component model predictions			
		0.2 Hz, 23°		0.05 Hz, 50‐80° or 23°	
		IO	RO	IO	RO
1_o_	279	168	277	‐	282
2_o_	22	145	39	NA	NA
3_o_	335	133	337	‐	354
4_o_	93	125	143	‐	137
5_o_	247	76	246	73	247
6_o_	52	229	39	NA	NA
7_o_	61	236	53	NA	NA

The “NA” marker indicates that unit was not tested at these conditions. Hyphen (‐) indicates that input was identified by model.

The orientations of dynamic and static inputs predicted by the model for four other (4/7) neurons that did not have typical STC properties were almost in the same plane (Table 3). In one cell these inputs were in the same direction (Unit #4_o_), while in three other neurons they were in opposite directions. This explains why these neurons did not have the typical STC responses at any frequency tested. RVO of experimentally determined and model‐predicted static otolith inputs were compared at 0.2 and 0.05 Hz (Fig. 6F). The slopes of the linear regressions were 0.97 for 0.2 Hz (*R* ^2^ = 0.972, *n* = 7, *P* < 0.001) and 0.86 for 0.05 Hz (*R* ^2^ = 0.976, *n* = 4, *P* = 0.006).

Thus, the two‐component model was sufficient to explain the dynamic/irregular and static/regular convergence in otolith‐only neurons across tested frequency. The STC properties of some otolith‐only neurons indicate the activation of dynamic otolith‐related input. Based on the presence of significant dynamic/irregular otolith input in some otolith‐only neurons, it was assumed that the dynamic otolith‐related input could be also responsible for STC characteristics in some canal–otolith neurons.

### Four‐component model predictions of the regular and irregular canal and otolith inputs in canal–otolith neurons

A four‐component model was used for 13 units that were tested at 0.2 and 0.05 Hz to determine whether it could predict the behavior across frequencies with a single set of parameters. The regular canal‐related input was predicted with high accuracy (*R* ^2^=0.984, *P* < 0.001) and the slope of fit of the linear regression was close to unity (0.98, Figure S1G). The slope of the experimentally determined and model‐predicted regular (static) otolith input was also highly statistically significant (*R* ^2^ = 0.956, *P* < 0.001) with a linear regression of 1.05 (Figure S1H). Thus, the four‐component model accurately fit the data obtained at all tested frequencies with a single set of parameters (Figure S1G, H). Furthermore, the predicted orientations of the regular canal‐ and otolith‐related inputs were more accurate than the predictions of regular canal‐related input for lower stimulus frequency (Figure S1E) and regular otolith input for higher frequency (Figure S1B) accounted by the two‐component model.

The predicted irregular canal input was statistically significant in four neurons and the predicted irregular otolith input in 11 neurons (Table 2). The irregular otolith input was not clustered in the planes of either the regular canal or otolith inputs.

The predicted regular canal‐related inputs were close to the orientation of the canal inputs measured experimentally (7°, 3 : 17°, *n* = 13). The difference in predicted orientation of the irregular canal inputs relative to their experimentally determined regular canal inputs was 37° (36.5 : 40°, *n* = 4). The predicted regular/static otolith inputs were also close to those measured experimentally (11°, 5 : 28°, *n* = 11). The irregular otolith inputs did not have specific orientation in the earth‐horizontal plane and were not clustered in the planes of either the regular canal or the otolith inputs; however, the difference in orientation of predicted irregular/dynamic otolith inputs related to the regular canal input was less (42°, 18 : 78°, *n* = 11) than to the regular/static otolith inputs (156°, 99 : 161°; *n* = 11).

In summary, predicted orientation of the regular canal input by the two‐component model varied from experimentally determined values of 0.2 Hz and 0.05 Hz by 8° and 13°, while the regular canal orientation predicted by the four‐component model for all frequencies was different from the experimentally determined values by 7°. Similarly, differences in regular otolith input orientation predicted by the two‐component model at 0.2 Hz and 0.05 Hz with experimental data were 41° and 15°, while the orientation determined by the four‐component model had a difference with experimental data of only 11°.

### Changes in STC responses due to orientation adaptation

#### Canal–otolith convergent neurons

Nine vertical canal–otolith neurons were tested before and after orientation adaptation (Unit #1‐9, Table 2). In five of these neurons, STC properties were determined at 0.05 Hz (Unit #2‐6). Only three neurons (Unit #4, 7, 8) had STC properties at 0.2 Hz.

Orientation adaptation shifted RVO of five neurons toward acceleration of gravity (Unit #1, 2, 4, 7, 8). In one other neuron, significant changes in RVO were opposite to the acceleration of gravity (Unit #9). In all six neurons, the shift of RVOs was by 30° (20 : 34°). In two additional neurons, there were no changes in RVO (Unit #3, 6). RVO after adaptation in Unit #5 was not determined (Table 2). The regular and irregular convergent inputs were predicted by the complex model in six adapted neurons that were tested at two frequencies. There was a close correlation between the experimental and model‐predicted orientations of the regular canal input (slope of linear fit was 0.96, *R* ^2^ = 0.981, *n* = 6, *P* < 0.001) and regular otolith input (slope of linear fit was 1.28, *R* ^2^ = 0.974, *n* = 5, *P* < 0.001) after prolonged head reorientation (Figure S1G, H, filled symbols). Thus, the four‐component model accurately predicted both regular inputs in every instance before and after adaptation.

**Figure 7:**
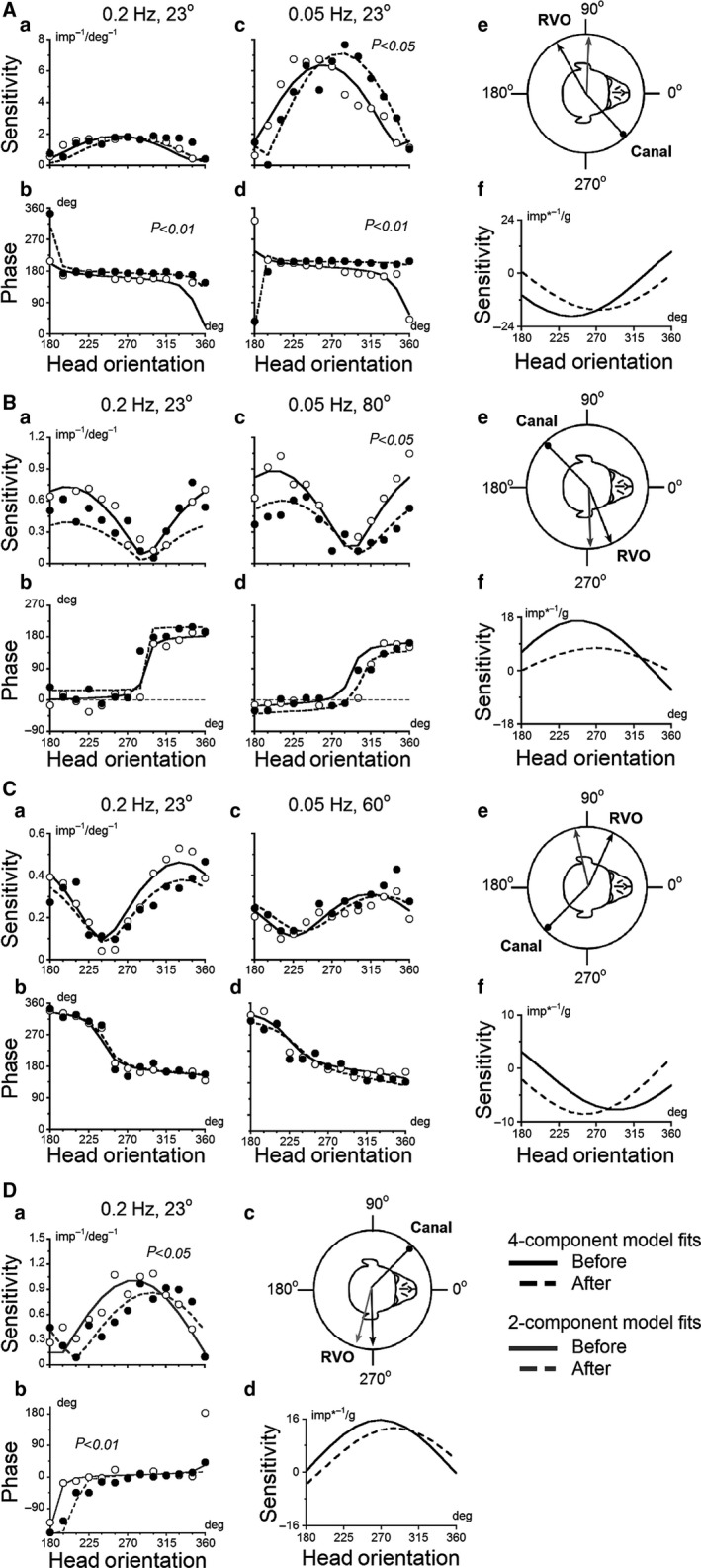
(A–D) Canal–otolith neurons (Units #1, #2, #4, #9) tested before (open symbols) and after (filled symbols) orientation adaptation. Sensitivities (A–C a & c, Da) and phases (A–C b & d, Db) plotted as head orientation in yaw plane (abscissa) during oscillations at 0.2 Hz and 0.05 Hz with different peak tilt velocities. Curves are the model fit through the data obtained before (solid lines) and after (dashed lines) orientation adaptation, with the four‐component model fit (black lines) and two‐component model fit (gray lines). (A–C f, Dd) RVO determined before (black solid curve) and after (black dashed curve) orientation adaptation. Note that experimentally determined canal‐related (black drumstick on inset above A‐C e, Dc) and otolith‐related inputs were closer to a single plane before (black arrow) compared to after (gray arrow) orientation adaptation re‐gravity for 2 h.

After orientation adaptation, the angle between the static otolith and the vertical canal inputs increased in two neurons (Unit #7, 9) and decreased in four other neurons (Unit #1, 2, 4, 8), and, therefore, STC characteristics could be altered due to changes in RVO after adaptation. For instance, after adaptation in Unit #1 the sensitivities and phases significantly changed consistent with shift of RVO (Fig. 7A). Before orientation adaptation, the neuron had no STC response at 0.2 Hz or at 0.05 Hz (Fig. 7A a–d, open symbols). The unit had regular canal and otolith inputs that were approximately collinear and oppositely directed, and angle between these inputs was 165° (Fig. 7A, inset). After orientation adaptation, the angle between canal and static otolith inputs experimentally determined was 133°. In this unit, the spatial gains (S) value to tilting and unit firing rate for upright head position were significantly decreased from 19.3 to 16.5 imp/s and from 39.1 to 25.9 imp/s (*P* < 0.05), respectively. Thereby, the contribution of static otolith input was decreased in final canal–otolith‐related responses during sinusoidal angular rotations at 0.05 Hz with peak amplitude of 23°. Thus, a significant shift of substantial changes of neuronal responses to the sinusoidal rotations was observed at 0.05 Hz (Fig. 7A c, d; filled symbols), consistent with significant change in static otolith sensitivity responses to tilts (Fig. 7A f). In another adapted canal–otolith neuron (Unit #2) shown in Figure 7B, the angle between regular canal and otolith inputs also was more orthogonal after adaptation, changing from 157° to 136°, and its STC responses (Fig. 7B a–d) accorded with change in spatial sensitivity for static otolith input (Fig. 7B e).

Three other units (Units #3, #6 (not shown) and Unit #5 in Fig. 4D–I) also did not change their RVO in yaw plane after orientation adaptation. However, some changes in STC responses were determined at 0.05 Hz after adaptation. Especially, the sensitivities of neuronal responses significantly decreased while the phases did not in Unit #5 (closed symbols Fig. 4F, I). The complex model predicted that orientation of regular/static otolith input in horizontal head plane was similar before and after adaptation. If spatial sensitivity value of static otolith input is increased or decreased, RVO can be aligned toward yaw head axis as a representation of head orientation relative to gravity. Similar sensitivity changes in RVO were described for otolith‐only neurons after reorientation in side‐down positions (Eron et al. 2009).

Another example (Unit #4) had STC characteristics at both frequencies before and after adaptation (Fig. 7C a‐d). Measured RVO of this unit was affected by orientation adaptation (Fig. 7C e). However, the phase changes as a function of head orientation in yaw plane were similar after adaptation (Fig. 7C b, d), while the sensitivities to rotations at 0.2 Hz have decreased (Fig. 7C a). The new model predicted that this unit had a rearrangement of irregular and regular otolith inputs that was responsible for the changes at 0.2 Hz. The model predicted that significant irregular otolith input had similar orientation in yaw before and after orientation adaptation (Table 2), however, the sensitivity value decreased from 0.24 to 0.15 imp/s, which can also be applied to development of STC behavior and its sensitivity changes at 0.2 Hz.

In two other neurons (Unit #7, 8), the RVOs were significantly altered after adaptation (*P* < 0.05). However, the STC properties at 0.2 Hz were similar before and after adaptation (not shown). The model predicted that these neurons could show STC behavior at 0.05 Hz, but no data are available at this frequency after adaptation. One other neuron (Unit #9) did not have STC responses at 0.2 Hz before adaptation (Fig. 7D). After orientation adaptation, RVO had shifted by 16° relative to the direction of gravity. As a result, the angle between the canal and otolith inputs changed from 134° to 150° (Table 2). This made the orientation of the two inputs more collinear and as a result, the unit did not acquire any STC properties.

#### Changes in otolith‐only neurons

Five otolith‐only neurons were also tested after orientation adaptation. The changes of RVOs were less than 16°. Orientations of experimentally determined and model predicted static otolith inputs were comparable before and after adaptation. Two neurons had STC properties before adaptation at 0.2 Hz. After adaptation, one neuron lost its STC properties at 0.2 Hz (Fig. 6A, filled symbols; *P* < 0.05, *F*‐statistic). The model predicted that the change in orientation of the dynamic otolith input was 55° (Fig. 6E). As a result, the angle between predicted dynamic and static otolith inputs widened from 109° before to 167° after adaptation, making two vectors collinear. Consequently, this neuron lost its STC characteristics (see open and filled symbols in Fig. 6A, B). STC characteristics of the second neuron were not affected by orientation adaptation (*P* = 0.158; F‐statistic), neither were there any significant changes in model‐predicted orientations of static/regular and dynamic/irregular input (Table 3). Input changes were 11° for static inputs and 2° for dynamic inputs; and angles between predicted dynamic and static otolith inputs were comparable before and after adaptation: 106° versus 115°, respectively.

In conclusion, model‐based analyses indicate that although orientation of the static otolith input is not affected by prolonged head side‐down orientation, the orientation of the dynamic otolith input could be due to orientation adaptation in some cells. Furthermore, the model predicts that orientation adaptation, previously demonstrated for regular/static otolith input (Eron et al. 2008a,b), could also occur in irregular otolith input.

#### Change in spontaneous neuronal response in otolith‐related neurons

Spontaneous neuronal activity for an upright head position significantly changed after orientation adaptation in five VO neurons and four otolith‐only neurons. The range of FR changes at rest for canal–otolith neurons was from 4.7 to 17.23 imp/s (with maximal change in Unit #2; 30.03 ± 2.16 imp/s before vs. 12.80 ± 2.24 imp/s after) and for otolith‐only neurons was from 4 to 66.1 imp/s (with maximal change in Unit #1_o_; 25.98 ± 0.92 imp/s before vs. 92.06 ± 1.97 imp/s after). Thus, static otolith sensitivity in some otolith‐related neurons can be altered in response to orientation adaptation.

## Discussion

In this study, about 42% of the vertical canal‐ and otolith‐related neurons and the otolith‐only neurons showed typical STC behavior at least at one of the frequencies. We demonstrated that the spatial characteristics of the majority of otolith‐only STC neurons tested at different frequencies could be adequately described by the two‐component model that assumed convergence of the static (regular) and dynamic (irregular) inputs, but only for particular frequency. This is consistent with original finding for otolith‐only neurons (Angelaki 1992a, 1993; Angelaki et al. 1992b; Bush et al. 1993; Angelaki and Dickman 2000). In our study, some otolith‐only neurons required an additional contribution of irregular input with different polarization vector to explain frequency‐related STC response changes. The two‐component model was still able to fit adequately data of canal–otolith neurons obtained at any particular frequency; however, the model predicted the different orientations of the static otolith polarization vector for various stimulus frequencies. The four‐component model assuming both regular and irregular otolith‐ and canal‐related inputs was able to explain experimental data for canal–otolith neurons at all tested conditions as a single set of data. The experimentally measured regular canal‐related and regular otolith‐related inputs were close to model‐predicted values.

As consistent with the previous reports (Baker et al. 1984b; Kasper et al. 1988), the orientation of regular otolith‐related input was more accurately predicted by two‐component model for lower stimulus frequency (at 0.05 Hz, Figure S1D and F vs. B), while the predicted orientation of regular canal input was closer to experimentally determined values at higher frequency (at 0.2 Hz, see Figure S1A vs. E). This occurs because the otoliths and vertical semicircular canals activated by the head oscillations about spatial horizontal axis, but the portion of FR modulated due to otolith activation increases at low frequency and larger angles of oscillation, while FR portion related to canal activation is modulated relative to peak stimulus velocity (Fernández and Goldberg 1971, 1976a,b; Goldberg and Fernández 1971a,b; Highstein et al. 1987; Goldberg 2000).

In contrast to the two‐component model, the four‐component model applied to the data tested at least two or several frequencies predicts orientations of the regular canal and regular otolith inputs and the obtained results very close to experimentally determined orientations of the vertical canal and static RVO (Figure S1G, H). The irregular/dynamic otolith input was predicted by the four‐component model in seven canal–otolith neurons, while the irregular canal input was predicted only in three neurons. While irregular canal input had a small contribution in the response of majority canal–otolith neurons, the contribution of irregular otolith input was substantial, like that of otolith‐only STC neurons. The regular and irregular otolith convergence on the canal–otolith VN neurons has been demonstrated previously (Angelaki and Dickman 2000; Newlands et al. 2017). Although the canal‐related input could be a significant contributor to STC response (Schor et al. 1984; Yakushin et al. 2006), our results indicate that the dynamic otolith inputs play a significant role in STC responses not only in otolith‐only but also in canal–otolith convergent neurons.

In some studies, the STC response in 2‐D were characterized by an ellipse model, which is equivalent to the definition of two response vectors in spatial and temporal quadrature, and characterized tuning ratio (TR) as minimum/maximum response sensitivity (Angelaki et al. 1992b; Angelaki 1993; Bush et al. 1993). Unfortunately, the ellipse model and TR have some limitations. For instance, if the static and dynamic vestibular convergent inputs are nearly aligned in central neurons (see for canal –otolith neuron in Fig. 5B g, and for otolith‐only neuron in Fig. 6E, gray arrows), the ellipse has collapsed to a straight line. It that case, in theory, the TR value achieves null; however, the real TR value could be calculated while the significant sensitivities for dynamic and static convergent inputs have been identified.

When the static and dynamic inputs are nearly aligned in same or opposite directions, the nonlinear interactions as summation or subtraction of convergent inputs from each other occur and depend on the angle between active convergent inputs. In a recent study in VN neurons that used the combinations of rotation and translation signals, a nonlinear interaction between these two sensory modalities was reported. The coincident peak responses were proportionally stronger than other, off‐peak interactions (Newlands et al. 2017). Thereby the neurons with the nearly aligned static and dynamic vestibular convergent inputs have STC responses which exhibit “cosine‐like” tuning with steady phase shifts between phases for the dynamic and static inputs. In the events when three or four vestibular convergent inputs are activated, the TR is also insufficient to characterize the STC behavior. Certainly, we understand and assume that the data fit provided by the four‐component model would not be necessary to be a complete fit at another set of parameters of testing, while the contributions of activated afferent inputs into a total weight of neuronal response could be different at other frequencies. According to many previous investigations (Baker et al. 1984b; Kasper et al. 1988; Angelaki et al. 1992b; Angelaki 1993; Bush et al. 1993; Dickman and Angelaki 2002; Angelaki and Dickman 2003; Zhou et al. 2006; Chen‐Huang and Peterson 2010; Yu et al. 2012) we propose that same central vestibular canal–otolith neuron with a complex convergent afferent inputs of both vestibular modalities and regularities discharges, and that has broadly tuned response and changed response vector (nonunitary) at different stimulus frequencies, should evince very sophisticated STC behavior. In other words, the same neuron may appear all sort of responses at different ranges of stimuli, namely: the non‐STC (cosine‐like tuning), typical STC (non‐cosine‐like tuning), non‐typical STC (cosine‐like tuning) responses, which can be specified by the various contributions of activated vestibular afferents according to their thresholds. It should be noted that a small set of amplitude‐frequency stimuli with the sinusoidal head rotations about earth‐horizontal axis was limited by an experimental daily protocol. Thus, the four‐component fitting model could be used to predict all activated convergent vestibular inputs in canal–otolith neuron even for a small set of stimulus frequencies, and this approach can be used in prolonged experiments with single neuronal recording, especially, when the total numbers of tests before and after adaptation are limited by the experimental procedures and routine.

In this study, the most of central vestibular neurons in VN had different types of convergences (Table 1). Especially, 39% (16/41) of the canal‐related neurons had inputs from two semicircular canals, 83% (34/41) of the canal‐related neurons were also otolith‐related, and 80% (33/41) neurons were vertical canal related and the most of them had also otolith‐related inputs (68%, 28/41). A quarter of all tested vestibular neurons (14/55) were otolith‐only neurons.

We did not find significant differences in spatial canal sensitivity for different sorts of canal‐related convergences. Particularly, the central canal–otolith neurons had comparable values of spatial sensitivity within 0.5–0.7 imp*s^−1^/deg*s^−1^. The spatial sensitivity to the head tilt for otolith‐only neurons is twice as much as for canal–otolith neurons. In the experiments with horizontal plane linear translations, the higher otolith sensitivity in otolith‐only cells was shown (Dickman and Angelaki 2002). This evidence may indirectly indicate that the density of afferent projections from the end‐organs onto the otolith‐only neurons is more substantial than for canal–otolith neurons. The predominant orientations of RVO in the horizontal plane for both classes of neurons were not found in our study.

Since we have disclosed previously that the static otolith RVO can be adaptively changed due to prolonged head reorientation re‐gravity (Eron et al. 2008a, 2009), we can verify the hypothesis that adaptive re‐orientation of otolith convergent inputs could induce the changes in STC response in central vestibular neurons. As a result, some canal–otolith neurons changed the convergent responses according to their RVO changes with appearing or disappearing of the STC behavior. Furthermore, the otolith‐only neurons may also change their STC responses due to the changes of spatial properties of either dynamic/irregular or static/regular otolith inputs. Previously, we have proposed that shift of RVO in 2‐D in the otolith‐related neurons are generated by changes in the weight of afferent inputs with different individual vectors which are differentially adapted when the head is held for prolonged periods of re‐gravity (Eron et al. 2008a, 2009). The magnitude of RVO shift in otolith‐only neurons was considerably less in contrast to canal–otolith convergent neurons, but the spatial sensitivity of some otolith‐related neurons could be change appreciably (Eron et al. 2009).

In our investigations, the RVO of static otolith input was plotted as a vector onto horizontal plane (in 2‐D); however, the increase or decrease in value of maximal spatial sensitivity to static tilt after orientation adaptation in otolith‐related neurons characterize the shift RVO relative to the vertical axis (in 3‐D). For instance, the orientation of regular/static otolith input in horizontal plane predicted by the complex model was similar before and after orientation adaptation in canal–otolith neurons shown in Figure 4; however, the STC response significantly changed for head oscillations at lower frequency indicating the decrease in spatial sensitivity (Fig. 4E and F) for static otolith input. While the orientation and spatial sensitivity of canal convergent input have been stable after orientation adaptation re‐gravity, such changes in STC behavior may occur if static RVO shifts toward *Z*‐axis.

The shift of total RVO after orientation adaptation also implies a presence of broadly tuned convergence from otolith afferents. Furthermore, the diversity between broadly and nearly tuned primary otolith afferents that differ in both their spatial and temporal response properties represents a mean of spatio‐temporal filtering (Angelaki and Dickman 2000). It is tempting to speculate that prolonged head reorientation re‐gravity might initiate a new interaction of low‐pass and high‐pass filters in single neurons during the frequency‐dependent processing of vestibular sensory information. Additionally, after orientation adaptation, the spontaneous activity during upright head position had been changed as well in some canal–otolith and otolith‐only neurons. We speculate that changes in threshold and response variability in the regular and irregular otolith afferents could also involve the changes in RVO and spontaneous FR to upright head position after prolonged head reorientation re‐gravity.

Perhaps, one of the most challenging questions is a physiological significance of STC in central vestibular‐only neurons in our daily life. We suggest that main role of STC processing in canal–otolith neurons is a link of two coordinate systems within the vestibular system, namely: external system (i.e., accelerations in spatial coordinates) and internal system (i.e., rotations in head coordinates); and the damage of STC processing could initiates difficulties in coding of vestibulo‐motor reactions at the level of brain stem and follow‐up difficulties or disorder in encoding of spatial orientation, perception, and spatial memory implemented at other brain levels/structures. It should be noted that vestibular‐only and vestibular‐plus‐saccade neurons encode indirectly a slow component of vestibulo‐ocular reflex (VOR) via velocity storage mechanism (Reisine and Raphan 1992; Yakushin et al. 2017), while a direct pathway of VOR is coded by position‐vestibular‐pause and eye‐head velocity neurons (Fuchs and Kimm 1975; Scudder and Fuchs 1992). It is shown that the adaptation of linear and angular VORs is specific to head orientation relative to gravity (Shelhamer et al. 2002; Yakushin et al. 2003a,b, 2005b). It is still unknown, however, how the network processing performed by the pure vestibular neurons (including canal–otolith and otolith‐only cells) occur to represent the gravity‐dependent component of VOR adaptation in secondary vestibulo‐oculomotor neurons in VN. A significant role of otolithic input in modulation of spontaneous nystagmus has been reported for human subjects; in particular, the spontaneous downbeat nystagmus becomes minimal when the patients have rested upright for 2 h, and the decrease in spontaneous downbeat nystagmus is less pronounced when patients lie down to rest in the prone or supine positions for 2 h (Spiegel et al. 2010). In space flight investigations it has been shown that the illusion of tilt perception was reinforced during in‐flight centrifugation and it was reinforced on entrance into microgravity (Clément et al. 2001). In the experiments with unilateral labyrinthectomy in monkeys, the decrease in sensitivity, increase in threshold, and alteration in orientation of best responses to sinusoidal linear translations in horizontal plane occurred in the vestibular nuclei, furthermore the phase of the neural response to sinusoidal translational stimulation in horizontal plane changed with unilateral labyrinthectomy (Newlands et al. 2014). Theoretically, all of these findings could be explained by the modification of the STC behavior in central canal–otolith and otolith‐only cells after partial loss of vestibular inputs for one or both vestibular modalities.

In summary, the weights of the regular and irregular vestibular afferent inputs to central canal–otolith neurons can be accurately predicted by the new four‐component model for a few stimuli based on their STC responses. As a result, this model could be used to simulate a neuronal activity profile for each central cell for a wide range of vestibular stimuli, when typical STC behavior appears and/or disappears. Thus, we have demonstrated that the orientation adaptation of their polarization vectors can generate or alter their STC properties and that the STC behavior is linked to the neural network responsible for contextual learning during gravity‐dependent adaptation.

## Conflict of Interest

None declared.

## Data Accessibility

## Supporting information




**Figure S1.** Comparison of experimentally estimated and model‐predicted orientations of regular vertical canal and regular/static otolith inputs in the canal–otolith neurons
