# Supplementary figures and images for: Adaptation of spatio‐temporal convergent properties in central vestibular neurons in monkeys
Source: Physiol Rep. 2018 Sep 3;6(17):e13750. doi: 10.14814/phy2.13750 (PMC6121125; doi:10.14814/phy2.13750)

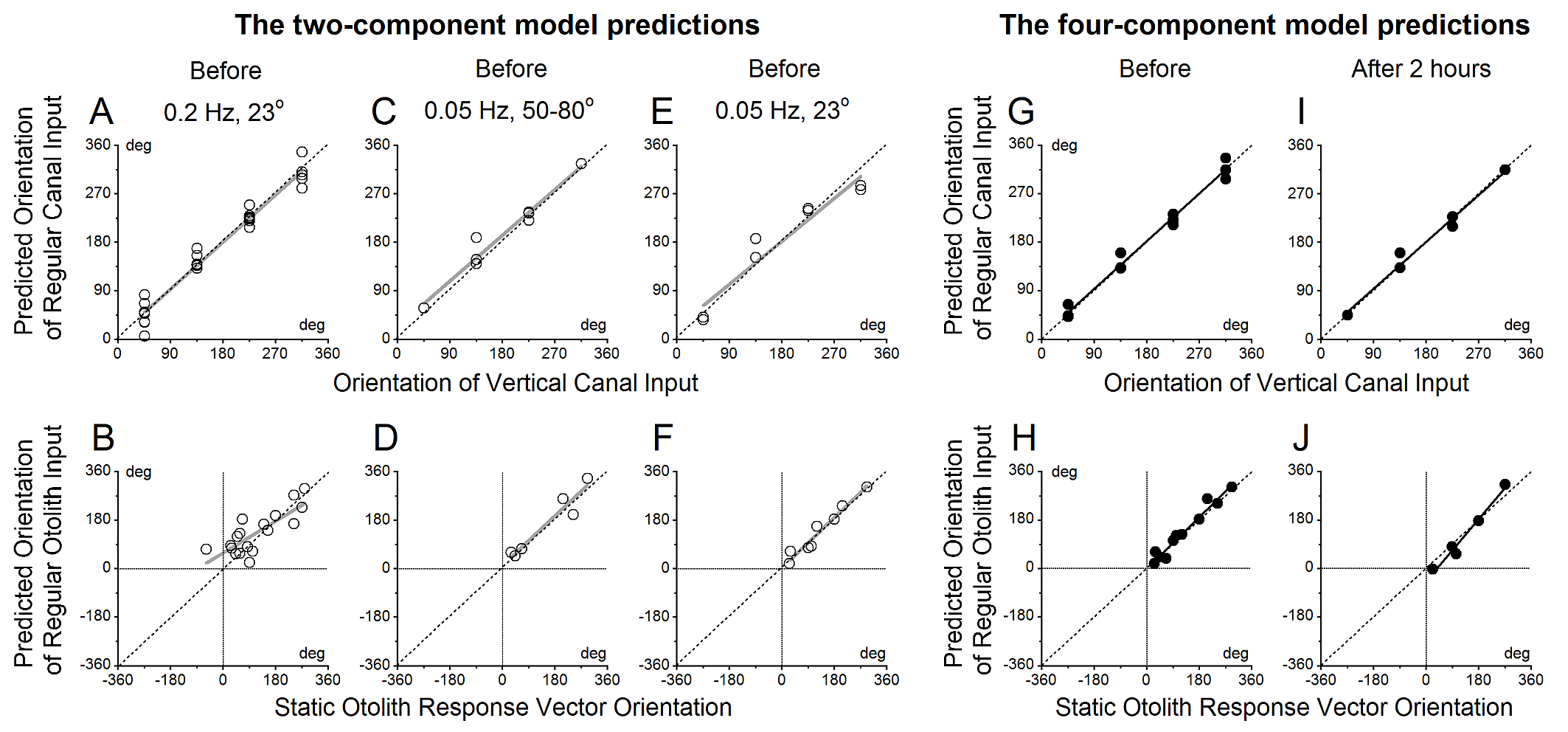

Supplement: Supplementary file 1 — Figure S1. Comparison of experimentally estimated and model‐predicted orientations of regular vertical canal and regular/static otolith inputs in the canal–otolith neurons [file PHY2-6-e13750-s001.tif]
